# Visual related quality of life after ICL V4c implantation in high myopia patients: a mini review

**DOI:** 10.3389/fmed.2025.1630025

**Published:** 2025-07-29

**Authors:** Huailan Zhao, Siquan Zhu

**Affiliations:** Department of Ophthalmology, Clinical College of Southwest Medical University, Luzhou, China

**Keywords:** high myopia, ICL V4c, visual-related quality of life, objective visual quality, subjective visual quality, complications

## Abstract

High myopia (≥ − 6.00 D) poses significant challenges to visual function and quality of life, with implantable collamer lens (ICL) V4c implantation emerging as a pivotal treatment. This mini review synthesizes evidence on visual-related quality of life (VRQoL) following ICL V4c implantation, focusing on predictability, objective/subjective visual quality, and complications. ICL V4c demonstrates long-term safety and efficacy, with safety indices (postoperative best-corrected visual acuity/preoperative best-corrected visual acuity) of 1.01–1.10 and efficacy indices (postoperative uncorrected visual acuity/preoperative best-corrected visual acuity) exceeding 0.90 over 5 years. Objective metrics, including higher-order aberrations and intraocular scattering, remain stable or improve postoperatively, while subjective outcomes show high patient satisfaction despite common but mild issues like halos (90.1% incidence) and glare (66.7% incidence). Complications such as cataract (1.7–6.8% incidence), corneal endothelial cell loss (≤5.7% over 8 years), and ICL malposition are rare and often manageable. While axial length progression in super-high myopia requires monitoring, ICL V4c remains a robust option for enhancing VRQoL in high myopia patients. Future research should prioritize large-scale, long-term studies to validate outcomes and optimize surgical protocols.

## 1 Introduction

Myopia, particularly high myopia (≥ − 6.00 D), poses significant challenges to visual function and quality of life, with global prevalence projected to affect 50% of the population by 2050 (1, 2). Myopia correction strategies primarily include corneal refractive surgery and intraocular refractive surgery. Intraocular approaches often involve lens-based interventions in the anterior chamber, with options including lens replacement and implantation (3). Direct lens replacement carries a higher risk of retinal detachment, limiting its clinical application. Corneal refractive surgeries, such as femtosecond laser small incision lenticule extraction (SMILE), are widely used for myopia correction, offering high safety, predictability, and stability. However, they are insufficient for treating high myopia and may cause postoperative complications like astigmatism and glare (4, 5).

The ICL V4c, an advanced posterior chamber phakic intraocular lens, features a 0.36-mm central aperture and bilateral openings, enhancing aqueous humor dynamics and reducing intraocular pressure fluctuations (6). This design minimizes optical quality impairment and cataract risk; for example, Lin et al. (7) reported no cataract formation in a 6-month follow-up of ICL V4c recipients. Constructed from biocompatible collamer material, the ICL V4c reduces complications such as anterior subcapsular cataracts and secondary glaucoma, with minimally invasive techniques further enhancing safety and tolerability.

Compared to traditional ICLs, the V4c model demonstrates superior clinical outcomes but is not without limitations. Chen et al. (8) reported postoperative glare in some patients, attributed to the lens design and surgical handling. Preoperative hydration of the lens surface is critical to maintain optical integrity during implantation, given the lens’s transparent, flexible, and compact nature, which requires meticulous handling to avoid contamination. The newer V5 model addresses these issues through preloaded implantation and an expanded optical zone, reducing glare and improving surgical efficiency (9).

*In vitro* studies by Pérez-Vives et al. (10) confirmed optimal optical quality with ICL V4c, while Chaitanya et al.’s (11) retrospective analysis of 67 myopic astigmatism patients (109 eyes) demonstrated significant refractive improvement: mean spherical equivalent reduced from −10.90 ± 3.7 D to −0.02 ± 0.13 D, and cylinder from −2.3 ± 1.3 D to −0.04 ± 0.2 D. Notably, 62% of eyes maintained stable best-corrected visual acuity, with only 1.8% requiring reoperation due to high vault complications.

With the proliferation of electronic devices, myopia prevalence and progression have become global public health concerns. The sixth-generation ICL V4c, a posterior chamber phakic intraocular lens with a central aperture, has emerged as a pivotal treatment for high myopia. Concurrently, the medical community’s focus has shifted toward postoperative quality of life. Visual-related quality of life (VRQoL), integrating objective metrics (e.g., higher-order aberrations, intraocular scattering) and subjective outcomes (e.g., uncorrected visual acuity), provides a comprehensive assessment of ICL V4c efficacy and safety. By bridging sociological, psychological, and clinical perspectives, VRQoL evaluations offer holistic insights into patient outcomes, guiding personalized interventions to enhance post-implantation quality of life. This review synthesizes current evidence on VRQoL after ICL V4c implantation in high myopia patients, aiming to inform clinical practice and future research.

## 2 Visual-related quality of life after ICL V4c implantation

In recent years, ICL V4c implantation has demonstrated prominent clinical advantages in correcting high myopia, featuring excellent efficacy, stability, and predictability, with minimal impact on postoperative VRQoL. VRQoL, a critical metric for evaluating refractive correction outcomes, is assessed through two dimensions: objective visual quality (wavefront aberrations, intraocular scattering) and subjective visual quality (uncorrected visual acuity).

### 2.1 Predictability and stability of ICL V4c implantation

Multiple studies confirm the long-term safety and efficacy of ICL V4c implantation, with safety indices (postoperative BCVA/preoperative BCVA) ranging from 1.01–1.10 and efficacy indices (postoperative UCVA/preoperative BCVA) exceeding 0.90 over extended follow-ups (12–14). The procedure maintains effectiveness in subgroups such as patients over 40 years old and those with shallow anterior chambers. Morkos et al. (15) further validated its utility in unilateral high myopia with anisometropic amblyopia, demonstrating stable visual acuity and refraction without long-term complications, thus positioning ICL V4c as a low-risk option that may help reduce the risk of amblyopia in selected pediatric cases.

The superior postoperative BCVA in most patients—equal to or better than preoperative values—may stem from the ICL V4c’s placement in the ciliary sulcus of the posterior chamber, which aligns with physiological optics, reduces the optical path to the retina, and enhances retinal magnification and image quality (16). However, myopic drift and axial length (AL) elongation are key factors affecting long-term efficacy. AL growth leads to myopia progression and UCVA decline, particularly in super-high myopia (≤−10.00 D), consistent with findings by Lee et al. and others (17, 18). Wang et al. observed that while efficacy indices remained above 1.00 at most time points, the 5-year index fell below 1.00, likely due to progressive myopia; higher preoperative myopia degrees correlate with greater progression risk. Chen et al. confirmed that myopia progression correlates with AL elongation rather than ICL type, underscoring V4c stability. Notable limitations include small sample sizes and short follow-up periods, necessitating longer-term data to validate durability.

### 2.2 Objective visual quality

#### 2.2.1 Wavefront aberrations

Objective visual quality encompasses wavefront aberrations and intraocular scattering. Wavefront aberrometry separates low-order aberrations (myopia, hyperopia, astigmatism) from high-order aberrations (HOAs; e.g., coma, spherical aberration, trefoil). Correcting low-order aberrations often induces new HOAs, which degrade VRQoL, making HOA assessment critical for refractive surgeries (19). High myopia, associated with AL elongation and corneal curvature changes, exacerbates optical issues (20). Clinically, ICL V4c reduces HOAs more effectively than excimer laser *in situ* keratomileusis (LASIK) (21), likely due to minimal corneal disruption, preservation of native corneal properties, and induction of negative spherical aberration. Comparisons between ICL V4 and V4c show no significant HOA differences at 3 months (22), while He et al. (23) noted similar HOA profiles and glare/halo severity between the two models. Collectively, these findings confirm superior HOA management with ICL V4c, enhancing objective visual quality.

#### 2.2.2 Intraocular scattering

Intraocular scattering, comprising forward and backward components, degrades VRQoL by reducing contrast through light veil formation on the retina, leading to halos, glare, reduced night vision, and contrast sensitivity (24). The Objective Scattering Index (OSI) correlates positively with scattering severity. Theoretically, ICL implantation—particularly the central hole—might increase scattering by complicating light paths, while lens rotation or residual astigmatism in toric ICL (TICL) patients could exacerbate this. Clinically, however, the stable positioning of ICL V4c in the ciliary sulcus minimizes tilt/shift, and its thin, hydrophilic collamer edges promote gradual refractive index transitions with aqueous humor, reducing scattering (25). He et al. (23) found no significant OSI changes post-ICL V4c, indicating neutral impact on intraocular scattering and preserving optical performance comparable to healthy eyes.

We have summarized the common objective visual quality indicators after ICL V4c implantation, see Table 1.

**Table 1:** Objective visual quality metrics after ICL V4c implantation for high myopia.

Assessment category	Key findings	Clinical implications	References
Wavefront aberrations	Significantly reduces higher-order aberrations (HOAs) compared to LASIK Induces negative spherical aberration, preserving corneal properties No significant HOA differences between ICL V4 and V4c at 3 months Stable HOA profiles with minimal glare/halo severity	Minimizes optical degradation, maintains retinal image quality Superior to corneal refractive surgeries in HOA management Central aperture design does not exacerbate HOAs	(21–23)
Intraocular scattering	Objective Scattering Index (OSI) remains unchanged postoperatively Collamer material and ciliary sulcus positioning reduce light scattering Central hole design avoids optical path disruption	Preserves contrast sensitivity and night vision Optical performance comparable to healthy eyes Minimal impact on visual clarity in low-light conditions	(23, 25)
Refractive outcomes	Mean spherical equivalent reduces from −10.90 ± 3.7 D to −0.02 ± 0.13 D Cylinder correction from −2.3 ± 1.3 D to −0.04 ± 0.2 D 62% of eyes maintain stable best-corrected visual acuity (BCVA)	Achieves near-emmetropic refraction in high myopia Effective management of myopic astigmatism Long-term stability supported by 5-year follow-ups	(11, 15)
Optical zone stability	Minimal tilt/shift of ICL V4c in ciliary sulcus Thin hydrophilic edges promote refractive index transition with aqueous humor V5 model reduces glare via expanded optical zone	Maintains consistent optical performance Minimizes light scattering from lens edge Design advancements optimize visual quality	(9, 25)

### 2.3 Subjective visual quality

#### 2.3.1 Visual acuity and contrast sensitivity

Uncorrected visual acuity (UCVA) improves significantly after ICL V4c implantation, with 5-year outcomes surpassing preoperative BCVA in most cases. Younger patients (<21 years) exhibit better contrast sensitivity, likely due to healthier refractive systems and fewer retinal pathologies (26). Long-term follow-up (8 years) by Kamiya et al. (27) showed 83 and 93% of eyes achieved refraction within ±0.5 D and ±1.0 D of target values, respectively, affirming durable visual stability.

#### 2.3.2 Visual quality questionnaires

Subjective outcomes are evaluated via validated tools like the Visual Function Questionnaire. Mohr et al. (28) reported that 90.1% of patients experienced halos, 66.7% glare, 60.0% visual fluctuations, 47.2% focusing difficulties, and 43.2% starbursts post-ICL V4c; while halos were most common, they caused only mild visual disturbance, with older patients more susceptible. This high incidence of halos may be attributed to the central aperture design of ICL V4c, which can alter light refraction patterns, especially in low-light conditions. Variations in such symptoms across studies may stem from differences in patient demographics (e.g., age-related lens transparency), surgical techniques (e.g., lens centration accuracy), and follow-up durations (e.g., symptom resolution over time). For instance, younger patients with healthier crystalline lenses might exhibit less severe scattering-related symptoms compared to older cohorts. Chen et al. (29) noted low glare severity despite high night-time glare scores, emphasizing the need to address poor night vision—a mechanism yet to be clarified. Patient satisfaction remains high: 5 years postoperatively, most reported improved self-image and confidence, with 50% attributing career advancements to ICL V4c. All patients cited enhanced daily convenience, particularly those with super-high myopia. Table 2 shows the Visual Quality Questionnaires for ICL V4c Implantation.

**Table 2:** Visual quality questionnaires after ICL V4c implantation.

Questionnaire type/assessment dimension	Key findings	Clinical significance	References
Subjective symptom prevalence	Halos: 90.1% incidence, mild visual disturbance Glare: 66.7% (predominantly nighttime), low severity scores Visual fluctuations: 60.0%, focusing difficulties: 47.2%, starbursts: 43.2% Older patients more susceptible to halos/glare	Common but non-disabling visual phenomena; central aperture design may contribute to halos but with minimal functional impact Night vision complaints require further mechanistic exploration	(29)
Patient satisfaction & quality of life	5-year follow-up: 90%+ report improved daily convenience 50% attribute career advancements to surgery Enhanced self-image and confidence in super-high myopia patients Younger patients (<21 years) show better contrast sensitivity	Objective refractive outcomes align with subjective well-being Surgical impact extends beyond visual acuity to psychosocial functioning Long-term satisfaction validates procedure durability	(26, 29)
Standardized questionnaire scores	Visual Function Questionnaire (VFQ) scores demonstrate significant improvement in Distance vision (85%+ within normal range) Role limitations due to vision (75% reduction in impairment) Social function and mental health domains	Quantitative validation of VRQoL enhancement Comprehensive assessment integrating visual and psychosocial metrics	(3, 29)
Night vision specific outcomes	High nighttime glare scores despite low self-reported severity Contrast sensitivity declines slightly in mesopic conditions but remains within clinical norms No significant correlation between OSI and subjective glare complaints	Discrepancy between objective scattering metrics and patient-reported glare Night vision optimization as a potential research priority	(23, 29)

### 2.4 Complications

Complications, including cataract, corneal endothelial cell loss, ICL V4c malposition, elevated intraocular pressure, and retinal detachment, are significant determinants of postoperative quality of life.

#### 2.4.1 Cataract

Cataract is a common complication following ICL V4c implantation, with risk factors including preoperative myopia > − 12.0 D, age >40 years, low vault, and increased preoperative lens thickness/density. Igarashi et al. (30) reported anterior subcapsular opacification (ASO) in 8 eyes (6.8%) after ICL V4c implantation, with 7 eyes (5.9%) developing ASO immediately postoperatively, likely due to contact between the lens anterior surface and ICL V4c during irrigation/aspiration. Long-term studies show that after 5 years, 2 eyes (2.11%) experienced BCVA loss of ≥2 lines due to peripheral ASO, and 1 eye (1.05%) due to central ASO. Compared to ICL V4, ICL V4c causes less lens impact and milder opacification, suggesting the central hole may nourish the lens, though it does not prevent ASO. The primary risk factor for ASO remains lens-ICL contact. An 8-year follow-up revealed cataract formation in 3 eyes (1.7%), with preserved visual acuity even in patients with preexisting mild peripheral ASO, confirming ICL V4c’s safety for high myopia.

#### 2.4.2 Corneal endothelial cell loss

Corneal endothelial damage after ICL V4c implantation may arise from direct contact with the corneal endothelium or postoperative inflammation, though corneal remodeling is also a proposed mechanism. Yang et al. (31) found high vault increases endothelial cell loss risk. Reported endothelial cell loss rates vary across studies: 1.6% ± 8.1% at 2.2 years, 2.68–3.87% at 5 years, 5.7% beyond 5 years, 2.60% at 7 years, and 3.6% ± 7.0% at 8 years, all within physiological loss rates. Even in patients with high vault (>1,000 μm), endothelial cell loss at 5 years was only 0.85%, demonstrating minimal long-term impact on corneal endothelium.

#### 2.4.3 ICL V4c position abnormalities

Postoperative ICL V4c positional issues include vault abnormalities, dislocation, rotation, and flipping, with vault decreasing over time. Li et al. (32) observed mean vault decreasing from 540.83 ± 186.13 μm at 1 month to 471.42 ± 211.35 μm at 2 years, with greater reduction in eyes with shallow preoperative anterior chamber depth (ACD) or high refractive error. High vault can be managed by ICL rotation; Fernández-Vega-Cueto et al. (33) and Wei et al. (34) showed vertical rotation reduces vault without requiring lens exchange. Notably, subcapsular opacification occurred in some normal-vault eyes, while low-vault eyes showed no opacification, indicating no linear correlation between vault and cataract risk. Standardization of safe vault thresholds requires further research.

#### 2.4.4 Elevated intraocular pressure (IOP) and glaucoma

Igarashi et al. (30) found no significant IOP elevation in 2.2-year follow-ups. Long-term studies (≥5 years) reported no pigmentary glaucoma, pupillary block, or vision-threatening complications. A 7-year study showed no IOP increase >20 mmHg or ≥5 mmHg from baseline, while Guber et al. (35) noted 13% incidence of elevated IOP over 7.3 years. The central hole design effectively prevents IOP elevation related to pupillary block or pigment dispersion.

#### 2.4.5 Retinal detachment

High myopia itself increases risk of rhegmatogenous retinal detachment (RRD) due to early vitreous liquefaction, posterior vitreous detachment, and lattice degeneration. Xu et al. (36) did not find increased RRD risk after ICL V4c implantation, though higher early RRD incidence within 1 year may relate to intraoperative disruption of ocular stability, IOP fluctuations, or surgical inexperience. Long-term data confirm ICL V4c safety for high myopia correction.

## 3 Conclusion

In conclusion, ICL V4c implantation represents a safe, effective, and stable option for correcting high myopia, with robust evidence supporting its long-term efficacy and favorable VRQoL. The procedure demonstrates predictable refractive outcomes, minimal impact on objective optical parameters like higher-order aberrations and intraocular scattering, and high subjective patient satisfaction, despite common but generally mild complaints such as halos and glare. While complications such as cataract, corneal endothelial cell loss, and ICL malposition exist, their incidence remains low and often within physiological or manageable ranges, particularly with advancements in surgical technique and lens design (e.g., central aperture, expanded optical zone in V5 models). Key challenges include monitoring axial length progression in super-high myopia to mitigate refractive regression and addressing rare but significant complications like retinal detachment. Future research should prioritize large-scale, long-term studies to validate durability across diverse populations and further optimize surgical protocols, ensuring ICL V4c remains a leading choice for enhancing visual function and quality of life in high myopia patients.
